# A Collaborative Approach for Surface Inspection Using Aerial Robots and Computer Vision

**DOI:** 10.3390/s18030893

**Published:** 2018-03-17

**Authors:** Martin Molina, Pedro Frau, Dario Maravall

**Affiliations:** Department of Artificial Intelligence, Technical University of Madrid, Boadilla del Monte, Madrid 28660, Spain; pedro.frauamar@gmail.com (P.F.); dmaravall@fi.upm.es (D.M.)

**Keywords:** Automated visual inspection, human-robot collaboration, surface inspection, aerial robotics

## Abstract

Aerial robots with cameras on board can be used in surface inspection to observe areas that are difficult to reach by other means. In this type of problem, it is desirable for aerial robots to have a high degree of autonomy. A way to provide more autonomy would be to use computer vision techniques to automatically detect anomalies on the surface. However, the performance of automated visual recognition methods is limited in uncontrolled environments, so that in practice it is not possible to perform a fully automatic inspection. This paper presents a solution for visual inspection that increases the degree of autonomy of aerial robots following a semi-automatic approach. The solution is based on human-robot collaboration in which the operator delegates tasks to the drone for exploration and visual recognition and the drone requests assistance in the presence of uncertainty. We validate this proposal with the development of an experimental robotic system using the software framework Aerostack. The paper describes technical challenges that we had to solve to develop such a system and the impact on this solution on the degree of autonomy to detect anomalies on the surface.

## 1. Introduction

The maintenance of certain infrastructures requires periodical inspections of surfaces (e.g., the surface of a dam, the facade of a building, an indoor wall, etc.) to find defects (e.g., holes, fissures, mould, spots, humidity, etc.) as symptoms of potential problems due to, for example, structural imperfections. Aerial robots can help human operators to inspect this type of surfaces. For example, operators can manually tele-operate aerial robots equipped with cameras to observe areas that are difficult to reach by other means.

In order to be more effective in this task, it is desirable for aerial robots to have a higher degree of autonomy. With the current state of technology, robots could perform automatically certain tasks related to surface inspection, but not with complete autonomy. Therefore, it may be more realistic to follow an approach based on a human-robot collaboration in which the operator delegates certain routine tasks to the robot, but the robot asks for assistance in the presence of uncertainty. In this domain, different techniques can be considered to provide autonomy to robots (e.g., path planning, computer vision, obstacle avoidance, visual alignment, coordination of multi-robot inspection, etc.). 

According to this, this paper presents a solution for visual inspection that increases the degree of autonomy of aerial robots following semi-automatic approach. In this work, computer vision techniques are used and extended to detect surface anomalies in a collaborative scheme. We validate our proposal with the development of an experimental robotic system. For the development of such a system, an existing software framework for aerial robotics called Aerostack was used, adding new components for visual recognition and user-system interaction.

The remainder of the paper is organized as follows. First, the paper describes the related work and presents how we consider the problem of surface inspection based on a collaborative work between human and robot. Then, the paper describes our approach for automated visual recognition of defects, using an existing method that we extended to be used with operator assistance. Next, the paper describes the software framework for aerial robotics Aerostack that we used to construct the robotic system, describing how we extended this framework with specific components for surface inspection. Finally, the paper describes a set of experiments that we performed to evaluate our solution. The experiments showed a good behavior for user-system interaction and visual recognition in the context of a collaborative surface inspection. As supplementary material, the paper provides the dataset of images from surfaces that we constructed and used to evaluate the quality of the visual recognition method.

## 2. Related Work

In general, the problem of automated visual inspection is a well-known specialized subfield of computer vision (for a general revision of methods and systems in this type of problem see for example references [1,2,3]). In automated visual inspection, different techniques in pattern recognition and image processing can be applied. This process usually includes image segmentation (e.g., by means of histogram-based thresholding or by means of edge-based filtering techniques plus heuristic post-processing) and the use of classifiers (e.g., statistical methods, kernel-based methods, neural networks, etc.). 

For example, computer vision techniques have been used in problems such as the following: atmospheric corrosion detection on petroleum plant equipment exposed to marine atmosphere [4], crack detection (which is a hard defect to detect in civil structures) [5] using Gabor filters invariant to rotation, and the inspection of surfaces of vessels [6] for rust detection using a magnetic robot crawler with a classifier ensemble (called PICARD) that combines different classification techniques (SVM, Bayesian classifier and random forest).

Our work deals with surface inspection by means of aerial robots. This type of robots have been recently used for different inspection problems, such as the inspection of buildings or large civil structures [7,8] (using techniques image reconstruction and monitoring), the inspection of power lines [9] (with solutions for the communication and collaboration of robot teams), inspection of marine vessels [10] (using visual-based recognition techniques for robot motion estimation and self-localization near the surfaces), fire detection [11] (using an heterogeneous fleet of aerial vehicles), or inspection of penstocks and tunnel-like environments [12] (proposing a self-localization method for this type of environment).

State-of-the-art technology in automated visual inspection has achieved high performance, but not the level of perfection required to perform inspection tasks in full autonomy. Therefore, we follow an approach based on using semi-autonomous aerial vehicles assisted by human operators. Our work can be considered an example on a novel paradigm in robotics systems with growing interest, namely, human-robot collaborative interaction [13]. This emerging approach is an answer to the trend to design more robust robotic systems by completing robot autonomy with online human intervention. This poses a huge challenge to robot system designers, since their robots must be able to operate as true partners with humans, which requires the implementation of the robot’s cognitive, communicative and perceptual capabilities that lie at the frontiers of the current state of the art of advanced robots.

The specific subfield addressed in this paper (vision-based human-robot collaborative inspection) is a novel research area about which we have found only few proposals in other domain problems. For example, Suzuki et al. (2000) [14] present an experimental prototype to cooperate with multiple teleoperated ground robots for inspection, and Kimura and Ikeuchi (1999) [15] use computer vision to recognize human motion while human and robot cooperate to assemble components.

## 3. Collaborative Surface Inspection with Aerial Robots

Surface inspection is a task that is usually done as part of the periodical maintenance work of certain infrastructures to detect symptoms of potential problems. In general, we consider that the goal of surface inspection is to explore a spatial area of a given surface in order to detect the presence of anomalies and classify them into prefixed categories. A simple example of this problem is to find imperfections such as fissures or holes in the surface of a wall. Other imperfections include spots, mould or humidity. 

A simple method to carry out this task with aerial robots is by teleoperation where the human operator manually guides a robot equipped with a camera. One of the immediate advantages of this approach is that it is possible to observe, with lower costs, areas that are difficult to reach by other means (e.g., extreme points on the surface of a dam or the surfaces of the pillars of a large bridge). 

In our work, we assume that there is an adaptive inspection in the sense that the environment conditions may affect the way the inspection is carried out. This means, for example, that a certain area may be difficult to be observed clearly during the inspection (due to bad illumination, excessive distance or another reason). Thus, it is not possible to determine in advance a fixed trajectory to be done to inspect the surface. Instead, it may be necessary for operators to manually teleoperate the drone to access new areas of interest during inspection and adjust the position or orientation (or turn on lights, etc.) to obtain better images of potential anomalies. 

In contrast to a fully manual teleoperation, it is possible to conceive a more supported method following a collaborative approach, in which the operator delegates certain routine tasks to the robot, but the robot asks for assistance in the presence of uncertainty. In this case, aerial robots perform certain tasks autonomously with less intervention of the human operator. This may contribute to perform safer operation (e.g., robots may be able to avoid obstacles that the operator does not perceive clearly from a distance such as overhead cables, vegetation, etc.), more accurate inspections (e.g., robots may generate automatically accurate trajectories to cover a surface to inspect), or more productivity by reducing the cognitive load of operators (e.g., robots can detect automatically the presence of certain surface anomalies).

**Figure 1:**
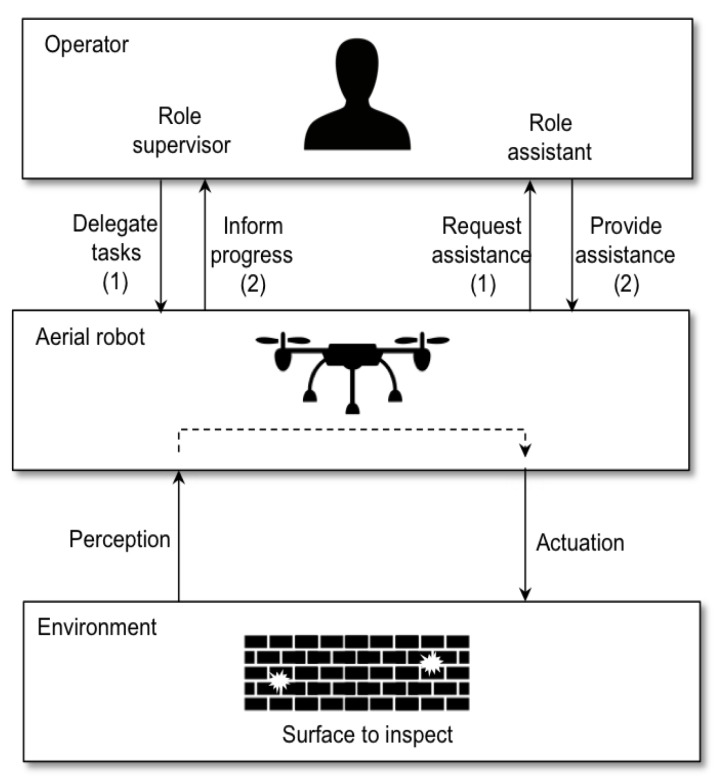
Human-robot interaction in surface inspection.

Figure 1 summarizes this type of human-robot interaction in which the operator can adopt two different roles corresponding to two interaction modes: supervised mode and assisted mode. The following sections describe in more detail how we understand such modes.

### 3.1. Supervised Mode

The supervised mode is related to the notion of supervisory control [16] in which a human operator is intermittently acting on the robot to delegate tasks. The robot closes an autonomous control loop through effectors to the environment. This concept has been used to design flexible interaction models, for example, for military mission planning of UAVs [17], swarming networks [18] or remote surveillance system [19].

To clarify the interaction modes, messages can be divided into categories according to the theory of speech acts [20,21,22]: assertive messages, which are sent to give certain information to the receiver (for example, the robot informs the operator the completion of a task), and directive messages, which cause the receiver to take a particular action. Within directive messages, it is possible to distinguish between two categories: action directives (requests for action) and information requests.

In the supervised mode, the operator sends action directives to the robot in order to delegate mission tasks. The operator may ask the aerial robot to perform an inspection mission, specifying the area to cover and the exploration strategy. In this case, the relation between operator and robot follows a hierarchical authority (as supervisor-subordinate schema) in which the operator delegates a set of tasks. 

During the development of the mission, the operator observes the robot behaviour and the robot sends assertive messages to inform about the mission execution progress (e.g., completed task or finished mission). These messages are useful for the operator to verify that the mission is being developed as expected. The operator can interrupt the mission under certain circumstances (for example, to avoid wrong behaviours). 

In this interaction mode, the robot shows autonomy to adapt to a dynamic environment while tries to reach its goal [23]. But the robot also shows autonomy to accept or reject the proposed actions according to characteristics of the environment and its own goals (e.g., safety goals) [24]. 

### 3.2. Assisted Mode

In the assisted mode, the human operator works as a resource for the robot, providing additional information. The robot may ask the operator questions as it works, to obtain assistance with perception and cognition. This allows the human to compensate for limitations of autonomy. This is related to the idea of collaborative control in which human and robot work together [25]. The human and the robot dialogue to exchange information, to ask questions, and to resolve differences. This interaction scheme is a kind of mixed initiative approach [26]. Both, the operator and the robot, may take the initiative of the conversation during the dialogue.

This interaction mode is required because robots have partial knowledge and are not completely self-sufficient. In this case, the robot works like the field technician (i.e., it is skilled, but may need help) and the operator is like the expert (i.e., she or he can provide assistance when needed) as it is considered in human collaborative control [27]. In the design of these systems, it is important to minimize the interaction time (i.e., the expected amount of time that a human must interact with the robot) [28].

In the particular case of inspection missions, robots may have recognition abilities for certain defects but, sometimes, certain defects are difficult to classify automatically. In addition, unexpected changes of the environment (e.g., shadows, wind, etc.) may require attention from the operator to decide the appropriate response. 

The interaction mode for assistance starts, for example, when the robot is not able to recognize the category (or it is uncertain about a selected category) of a detected defect in the surface. In this case, it sends an information request to ask the operator for the category of the detected defect. The operator answers the category or rejects the detection. In addition, the operator may help the robot proposing motion actions to have better views of the surface.

This interaction mode may also start when the robot is not able to complete a requested task because there is a problem in the environment such as: low visibility, low battery, lost position, high vibrations, impassable barrier, or unstable ground. This includes also the time out event that happens when it is not able to complete the task in the expected time due to unknown reasons. The operator helps the robot saying how to respond to these events. 

In general, it is also possible to consider that the robot may delegate certain specialized tasks to other robots. For example, the robot can transfer part of the mission to other robot because it does not have enough battery charge, or it can delegate a certain specialized task that require specialized actuators (e.g., use a special device to mark the detected defect on the wall).

## 4. On-Board Camera for Surface Inspection

**Figure 2:**
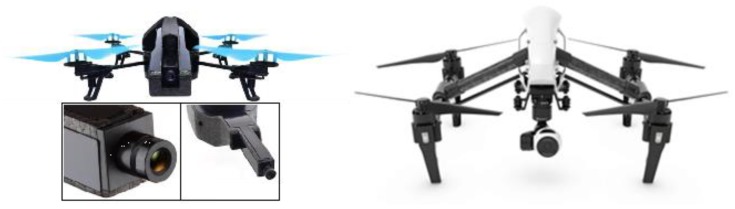
Commercial drones with on-board cameras: Parrot AR.Drone 2.0 (Parrot, Paris, France) (**left**) and DJI Inspire 1 (DJI, Shenzhen, China) (**right**).

In this work, we consider that the inspection task is performed with the help of a drone with a camera on board. This is a type of technology for which there are different types of available solutions in the market. For example, there are commercial aerial platforms, equipped with cameras, with characteristics that may allow inspection of surfaces. In some of the experiments described in this paper, we used Parrot AR.Drone 2.0 (Parrot, Paris, France)—a platform with an on-board front camera (Figure 2)—a flight time of about 35 min (power edition) and Wi-Fi communication with a maximum range of approximately 50 m. Table 1 shows examples of other general-purpose commercial platforms with different features that could be selected, for example, to obtain better images or to use bigger communication range.

Other robotic systems for inspection can be used with more specific designs, combining advanced platforms (e.g., DJI Inspire 1 Pro or Yuneec Tornado H920, DJI, Shenzhen, China) with better quality cameras (e.g., Zenmuse X5, DJI, Shenzhen, China; CGO4, Yuneec, Jiangsu, China; etc.) and mechanical camera stabilizers (gimbals).

### On-Board Camera

An on-board camera oriented to the front of the aerial vehicle can be used to inspect vertical and flat surfaces. The specific features of the camera will depend on the desired performance of the inspection task. For example, the resolution of the camera to be used will depend on the minimum size of the anomalies to be detected. Cameras in commercial drones normally use a CMOS image sensor with a resolution that may range for example from high definition HD with 1280 × 720 pixels (AR.Drone 2.0, Parrot, Paris, France) to ultra-high definition UHD with 4096 × 2160 pixels (Phantom 3 Pro, DJI, Shenzhen, China or Yuneec Typhoon H, Yuneec, Jiangsu, China). 

**Figure 3:**
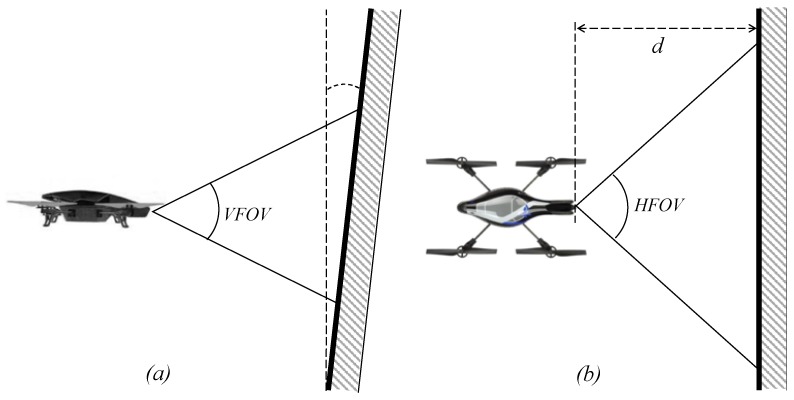
Location of the air vehicle with respect to the surface. The figure shows the vertical field of view VFOV of the camera (**a**), the horizontal field of view HFOV (**b**), vertical surface inclination ω and distance of the surface *d*.

The minimum size of the anomalies to be detected (represented here by horizontal length $l_{h}$ and vertical length $l_{v}$) can be estimated using the resolution $R_{h}\times R_{v}$ (horizontal × vertical) and the following two equations:

(1) $$l_{h}=\frac{r}{R_{h}}2d\tan\left(\alpha \right)$$

(2) $$l_{v}=\frac{r}{R_{v}}2d\frac{\tan\left(\beta \right)}{\cos\left(\omega \right)}$$

where *d* is the distance from the camera lens to the surface to be inspected, α = *HFOV*/2, β = *VFOV*/2 (*HFOV* and *VFOV* are respectively the horizontal and vertical field of views), ω is the vertical inclination of the surface, and *r* is a parameter that represents the minimum number of pixels needed to recognize a defect (in practice, it is possible to follow the general rule of thumb *r* = 10 or *r* = 20). The surface to be inspected may present certain vertical inclination (Figure 3). Considering that the front camera is fixed, if the value of inclination ω increases, then pixels represent more distance in the surface and, therefore, the minimum vertical length of anomalies to recognize increases, as it is established by Equation (2). 

When inspecting surfaces, it is useful to be able to fly at a short distance from the surface in order to detect smaller anomalies. This distance must be established taking into account safety requirements (based on the stability of the aerial platform and the environment conditions). For example, in our experiments, we used distance values of 50 cm < *d* < 150 cm.

In order to scan the complete surface, the vehicle can develop automatically a flight trajectory. This is an important advantage compared to manual inspection, since more precise trajectories can be automatically developed in large surfaces, preventing gaps from remaining uninspected zones due to drifts and other errors caused by the human operation.

**Figure 4:**
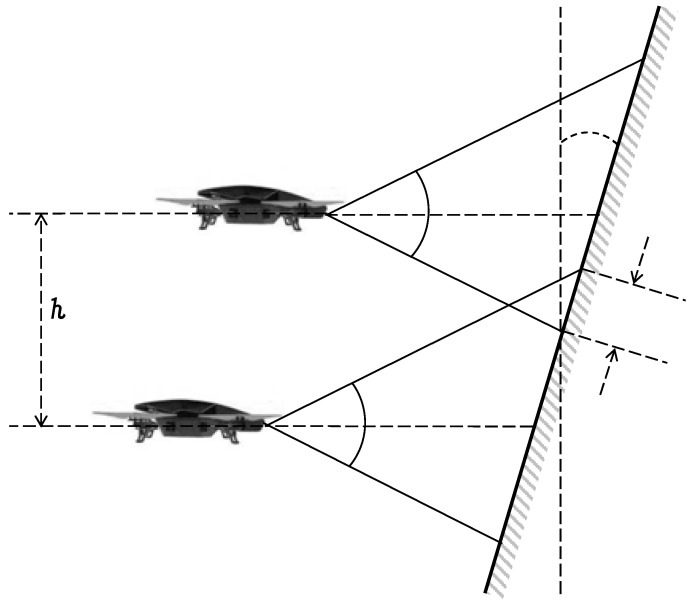
Vertical distance *h* between consecutive horizontal scans.

The specific waypoints that define such trajectories must be planned before the flight (for example, using the software tool Aerostack), considering the field of view of the camera. In the case of a horizontal inspection, the movement of the drone develops a sequence of adjacent horizontal scans, changing the altitude at the end of each scan. The difference in altitude between two adjacent horizontal scans (value *h* in Figure 4) depends on the vertical field of view and a small overlap between two adjacent scans (represented with parameter *g* that is manually adjusted for each particular case). This overlap is recommended to avoid gaps and it is also useful when edges of the photo are too distorted. To estimate the value of *h*, the following equation can be used:

(3) $$h=\left[d\left(\frac{\sin(\beta )}{\cos\left(\omega +\beta \right)}+\frac{\sin\left(\beta \right)}{\cos\left(\omega -\beta \right)}\right)-g\right]\cos\left(\omega \right)$$

Other considerations about the on-board camera can be taken into account for an adequate inspection performance. For example, the camera captures images that may be blurred due to vehicle movement This can be reduced with the help of mechanical gimbal stabilization (e.g., as it used in DJI Phantom 3 Pro) or digital imaging stabilization (Parrot Bebop 2), although the digital solution loses resolution. Camera optics may also generate distorted images that affect the inspection process. For example, AR. Drone 2.0 uses a wide-angle lens that produces a certain distortion that affects how our algorithm spatially locates the position of anomalies. To reduce this problem, it is possible to increase the overlap between adjacent scans (which increases the required number of scans). It may be also possible to use algorithms to automatically correct this distortion or to use lens cameras that do not produce distortion (e. g., the DJI Phantom Pro camera).

## 5. The Visual Recognition Method

### 5.1. The Method for Defect Recognition

In order to detect defects in still images of surfaces, we selected and implemented one of the existing methods in the literature. This method was based on the approach of frequency histogram of connected elements (FHCE) [29], which is a generalization of the conventional image histogram aimed at detecting and segmenting textured surfaces. This approach was selected for our problem due to its efficiency for image segmentation as it has been proved in practical problems such as wooden pallets [30] and road segmentation for autonomous car driving [31]. 

This approach uses the concept of neighbourhood. For a given pixel (i,j) of an image, its neighborhood is formed by a set of pixels which distances to (i, j) are not greater than two integer values r,s and is defined as $\varphi_{\left(i,j\right)}^{r,s}$. A connected element is the neighborhood selected such as the intensity *I* of a pixel (k,l) is a subset of a given grayscale range [T−ε, T+ε]:

$$C_{\left(i,j\right)}\left(T\right)=\varphi_{\left(i,j\right)}^{r,s}:\;I\left(k,l\right)\subset \left[T-\varepsilon ,\;T+\varepsilon \right],\;\forall \left(k,l\right)\in \varphi_{\left(i,j\right)}^{r,s}$$

Given the previous definitions, *H*(*T*) is defined as the sum of all the connected elements for each pixel of an image on different grey levels *T* where *T* is greater than 0 and inferior than the maximum intensity minus one:

(4) $$H\left(T\right)=C_{\left(i,j\right)}\left(T\right)\;0\leq T\leq I_{max}-1$$

Algorithm 1 summarizes the main steps of the version the FHCE method implemented to recognize anomalies on the surface. The first line of the algorithm is the identification of the interval of grey values [*k*_1_, *k*_2_] of an anomaly, which is determined by the analysis of the frequency histogram. This analysis can be done dynamically for each image or, in certain domains with similar defects, this could be done in advance and use the same values of *k*_1_ and *k*_2_ for all the images.

**Figure 5:**
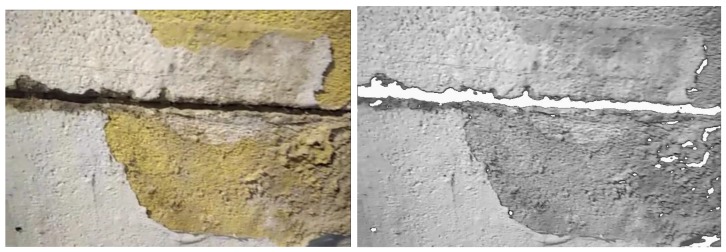
Example of original versus resulting image after flaw separation.

The next lines in the algorithm (lines 2–4) assign to each pixel a new distinctive colour *s* (e.g., white colour) if its grey value belongs to this interval. As a result, an image is obtained with the flaws separated from the background (Figure 5).
**Algorithm 1.** Defect recognition.**Input:** digital image *I* (a still picture)
1.[*k*_1_, *k*_2_] ← interval of grey values for anomalies (based on histogram analysis)2.**for each** pixel (*i, j*) **in** input image *I*
3.  *g_ij_* ← grey value of pixel (*i, j*) 4.  **if** (*g_ij_* ∈ [*k*_1_, *k*_2_]) **then**5.    substitute the value of pixel (*i, j*) by the distinctive colour *s*6.*R*_1_ ← rectangles obtained as contour of images of colour *s*
7.*R*_2_ ← rectangles *r_k_* ∈ *R*_1_ such as *area*(*r_k_*) ∈ [*k*_3_, *k*_4_]8.*D* ← classified rectangles *r_k_* ∈ *R*_2_ according to shape conditions9.**return**(*D*)

Then, the algorithm applies a heuristic approach to identify categories of imperfections. The last lines of the algorithm (lines 5–7) obtain the contour of the images of distinctive colour *s* as a rectangle and selects rectangles whose area *a* ∈ [*k*_3_, *k*_4_]. This constraint assumes that only imperfections of certain size are considered and, therefore, the wall must be observed from an appropriate distance. 

**Figure 6:**
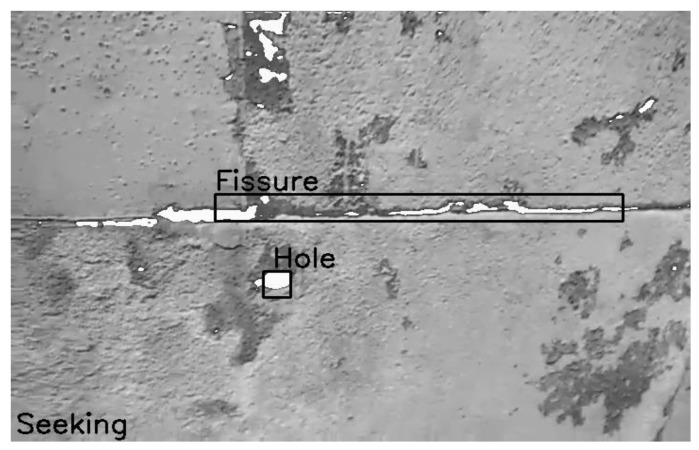
Example of image classification.

The method finally classifies the selected rectangles into categories according to their shape. For example, if *l*_1_ and *l*_2_ are the length of the sides in pixels, a rectangle with square shape, which satisfies that |*l*_1_ − *l*_2_| < 30 pixels, can be considered as a hole. Fissures can be characterized as a linear flaw, which is why the method searches for rectangles along a certain linear direction. As a result, the method obtains delimited areas for both flaw types (see Figure 6).

### 5.2. Defect Recognition with Operator Assistance

This section describes our solution to extend the previous method in order to be used in the collaborative approach presented in this paper. In general, the information recorded by a robot camera is received as a stream of still pictures (frames) at a certain frequency. For example, the aerial vehicle AR Drone 2.0 uses a front camera with a frame rate of 15 or 30 frames per second. The practical experience using robot cameras shows that the visual recognition usually presents gaps in the classified categories of the video stream, with some intermediate frames where the categories are misclassified.

In our approach, the presence of these gaps is used as a way to estimate a credibility value associated to the recognized category. This credibility represents the robot’s confidence in the recognition, and it may be a solution to decide when the robot may need operator assistance. We assume that a classification with a high credibility value corresponds to a sequence of classifications in the video stream with narrow gaps. On the other hand, a classification with low credibility presents wider gaps.

To implement this, our method uses a sliding window technique to analyse the video stream. The method uses a window size of *n* frames from the video stream. At each step, the content of this window is a frame sequence *F* = {*f_i_*}, an ordered set of frames *i* = 1, …, *n* where |*F*| = *n* is the cardinality of this set.

The uncertainty associated with an anomaly *a_j_* is represented with the credibility *Cr*(*a_j_*) ∈ [0, 1]. To obtain this value, the relative frequency of the detection of anomaly *a_j_* in the set of images *F*, is computed using the following equation:

(5) $$Cr\left(a_{j}\right)=\frac{n_{j}}{n}$$

where:

$$\begin{array}{c} n_{j}=\overset{n}{\underset{i=1}{\sum }}\delta (f_{i},a_{j})\\ \delta \left(f_{i},\;a_{j}\right)=\;\{\begin{array}{l} 1\;if\;anomaly\;a_{j}\;is\;detected\;in\;frame\;f_{i}\\ 0\;otherwise \end{array} \end{array}$$

The credibility is used to decide when the robot needs to ask operator assistance. For this purpose, our method uses an interval defined by two parameters [*k*_5_, *k*_6_] that establishes the uncertainty range when the robot requires assistance. Thus, the robot asks for operator assistance to confirm the recognition of anomaly *a_j_* when the credibility satisfies *Cr*(*a_j_*) ∈ [*k*_5_, *k*_6_]. The robot rejects (or confirms) automatically the recognition of anomaly *a* when *Cr*(*a_j_*) < *k*_5_ (or when *Cr(a_j_*) > *k*_6_) without asking for operator assistance.

Algorithm 2 summarizes this process. This algorithm uses the procedure described in Algorithm 1 to detect defects in still pictures (line 3). The recognized defects in the frames are collected to compute finally their credibility (line 8) using Equation (4). Each anomaly is automatically confirmed if its credibility is greater than *k*_6_ (line 10) or, if the credibility is greater than *k*_5_, a confirmation is requested to the operator (line 11). Otherwise the anomaly is discarded.

The values of the interval [*k*_5_, *k*_6_] need to be calibrated to have an adequate performance of the method. In general, if we increase *k*_6_, then the number of false positives decreases, and if we decrease *k*_5_, the number of false negatives decreases. However, very low values of *k*_5_ or very high values of *k*_6_ increase significantly the number of requests for operator assistance. For example, with the extreme values *k*_5_ = 0.0 and *k*_6_ = 1.0, all the detected anomalies must be confirmed by the operator. As Section 6 describes (experimental evaluation), we use parameter values such as *k*_5_ = 0.2 and *k*_6_ = 0.5 with a good performance in our domain. But our approach is general to accept other values according to the needs of certain applications or other visual recognition methods.
**Algorithm 2.** Defect recognition in a frame sequence with operator assistance.**Input:**
*F* ← {*f_i_*} ordered set of the last *n* frames of a video stream
1.*A* = ϕ, *R* = ϕ, *n*(*a_j_*) = 02.**for** (*i* = 1, …, *n*)3.  *D* ← {*a_j_*, *a_j_* ∈ **defect_recognition**(*f_i_*)} *// Algorithm* 14.  **if** (*D* ≠ ϕ) **then**5.    *A* = *A* ∪ *D*
6.    **for** (*j* = 1, …, | *D* |) *n*(*a_j_*) = *n*(*a_j_*) + 17.**for** (*j =* 1, …, | *A* |)8.  *Cr*(*a_j_*) = *n_j_* / *n // Equation* (5)9.  **if** (*Cr*(*a_j_*) > *k*_6_) **then** *R* = *R* ∪ {*a_j_*}10.  **else if** (*Cr*(*a_j_*) ≥ *k*_5_) **then**
11.    **if** (**operator_confirm_anomaly**(*a_j_*)) **then** *R* = *R* ∪ {*a_j_*}12.**return**(*R*)

## 6. The Aerostack Framework

To implement the collaborative approach for surface inspection described above in a robotic system, it is necessary to build a complex control architecture that integrates both reactive low-level components (e.g., for perception and motion control) and deliberative high-level components (e.g., for operator interaction). For this development, we have used Aerostack (http://www.aerostack.org) [32,33].

Aerostack is an open-source software framework that helps developers design and build the control architecture of aerial robotic systems, integrating multiple heterogeneous computational solutions (e.g., computer vision algorithms, motion controllers, self-localization and mapping methods, planning algorithms, etc.). Aerostack is useful for building autonomous aerial systems in complex and dynamic environments and it is also a useful research tool for aerial robotics to test new algorithms and architectures.

**Figure 7:**
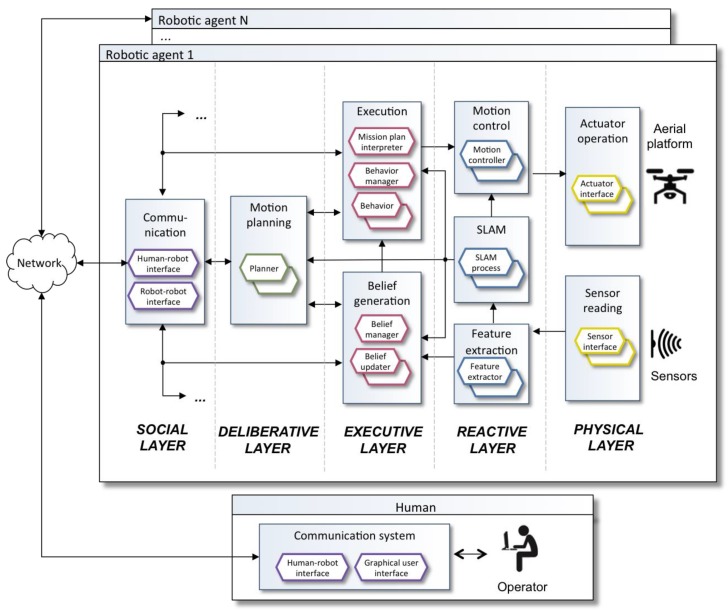
The multi-layered architecture of Aerostack.

Figure 7 shows the main functional components of the architecture. This design follows the hybrid reactive/deliberative paradigm, that is, an architecture that integrates both a deliberative and reactive approaches [34]. The design includes several layers: physical, reactive, executive, deliberative and social. The three layers reactive-executive-deliberative are based on the popular hybrid design known as the three-layer architecture [35]. The architecture includes also a social layer with communication abilities, as it is proposed in multiagent systems and other architectures with social coordination (e.g., [36]).

Aerostack (version 2.0) follows a behaviour-based approach which has been traditionally used in robotics [37,38,39]. A behaviour provides abstraction, hiding low level technical details and complexity, and it is appropriate to simplify how to specify complex missions. Aerostack provides a library of behaviours including basic motion behaviours (take off, land, keep hovering, keep moving, go to point, rotate, etc.) that can be used for surface inspection besides others (follow object image, self-localize by visual markers, wait, etc.). This is an open library that can be extended with additional behaviours to provide additional capabilities. A new behaviour can be included in the library by adding new processes. Aerostack uses ROS (Robot Operating System, http://www.ros.org). A new process can be created by programming a new ROS node in C++. The node may be integrated in the library by using inter-process communication mechanisms provided by ROS (e.g., publish/subscribe topics or service calls).

The operator can specify a mission plan in Aerostack using three alternative options: TML language, Python language or with behaviour trees. For example, the TML language [40,41] uses a task-based approach with hierarchies of tasks in which groups of behaviours are activated. Aerostack coordinates the execution of multiple behaviours avoiding conflicts when incompatible behaviours are requested to be active at the same time. 

**Figure 8:**
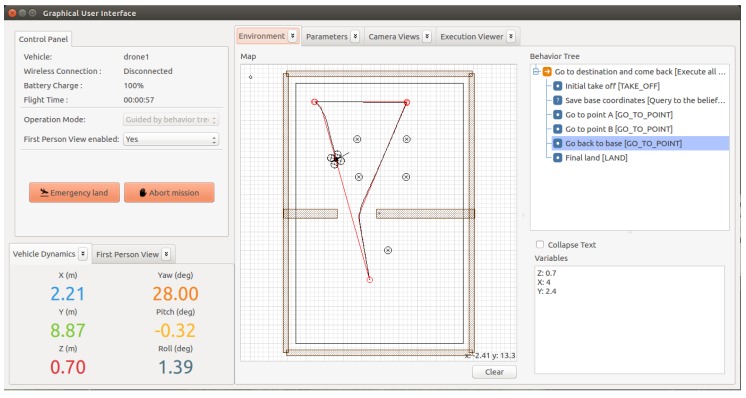
Example of screen presented by the graphical user interface of Aerostack to execute and supervise a mission plan.

Aerostack provides a graphical user interface (Figure 8) with which the operator can delegate tasks to the robot and the robot sends information to the operator to inform about its progress (this interaction mode corresponds to what we have called in this paper supervised mode). For example, the operator can use this interface to start the execution of a particular mission using the control panel. Once the mission has been initiated, the operator can use the interface to observe dynamic information (e.g., position coordinates and orientation), the location of the robot in an environment map with the existing obstacles, the camera image, and other information (parameter values, active behaviours, etc.). In addition, the graphical user interface includes buttons to help the operator to interrupt the mission execution (e.g., emergency land or abort mission).

**Figure 9:**
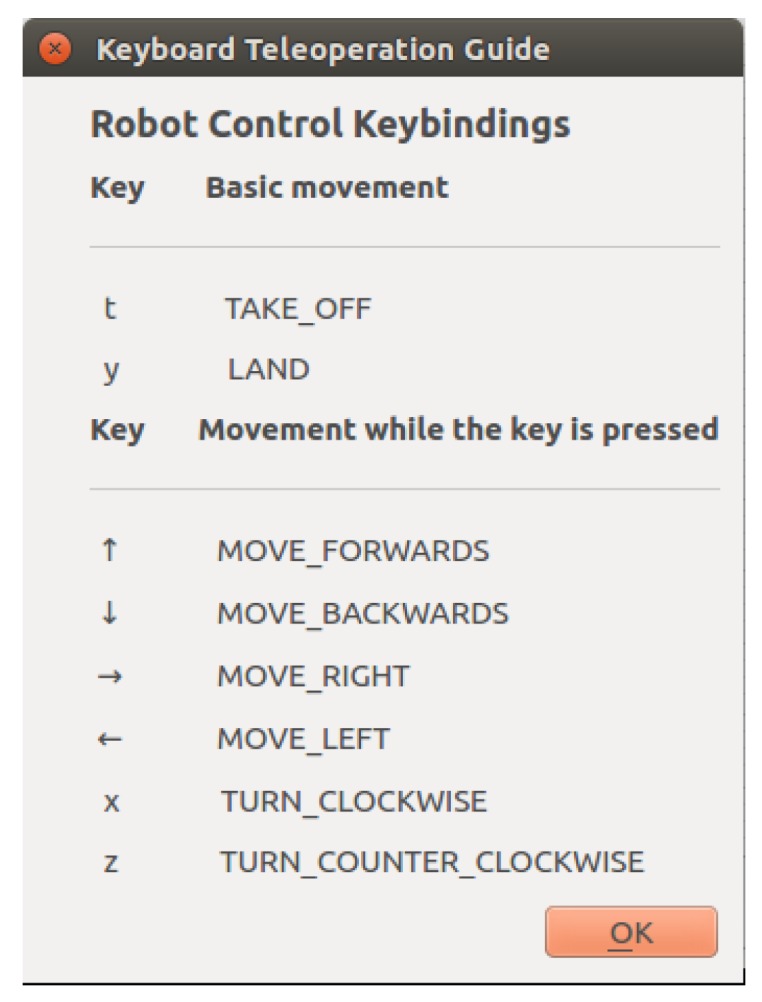
Available commands for tele-operation using the keyboard.

The operator can also use the keyboard to tele-operate the robot by executing certain basic motions (Figure 9), such as take-off, land, move forwards, turn clockwise, and so forth. These actions activate the corresponding behaviours to perform the motions.

## 7. Extensions for Surface Inspection

Aerostack provides a number of useful solutions for our surface inspection problem, but there are specific functionalities that are not covered. In particular, Aerostack does not provide two capabilities: (1) recognize automatically surface imperfections and (2) support the assisted mode in human-robot interaction, as it is required in our collaboration approach. This section describes how we extended Aerostack with additional components to cover such capabilities. 

Basically, the extension for surface inspection was done by adding new behaviours. In addition, it was necessary to modify an existing Aerostack component (the behaviour coordinator) to facilitate the support for assisted mode. Part of these changes are integrated in Aerostack (www.aerostack.org) to be available as open-source in the software framework.

**Figure 10:**
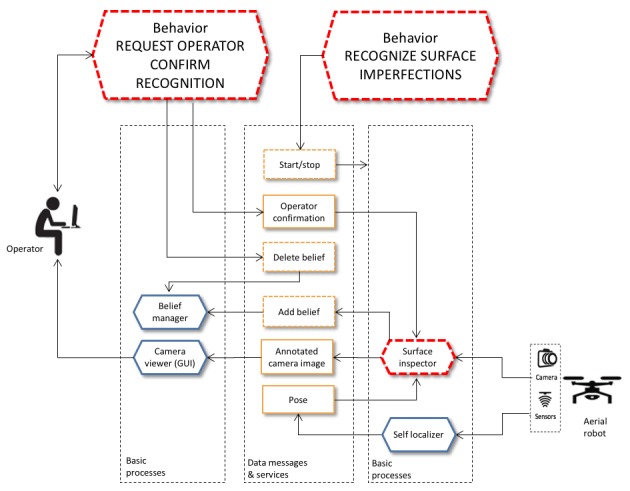
Example processes developed to support surface inspection (marked with a red dotted line).

Figure 10 shows new processes that were developed (marked with a red dotted line) and how they were integrated with other Aerostack processes. For example, the behaviour called *recognize surface imperfections* controls the execution of a specialized process called *surface inspector* that uses the computer vision technique presented in this paper to recognize automatically surface imperfections. This process receives an input image from the front camera together with the estimated pose of the robot and, as a result, generates the following information when an imperfection is detected: (1) the characteristics of the detected anomaly (category and position) that is stored as a symbolic belief and (2) the camera image with added marks indicating where the recognized defects are located.

Other processes specialized in human-robot communication were developed to support the assisted mode. Figure 10 shows a process that supports the behaviour called *request operator confirm recognition*. This behaviour controls the interaction with the operator in order to confirm or reject the recognized imperfection. The following section describes in more detail the specific techniques used to support the assisted mode.

### Assistance Mode in Human-Robot Interaction

As described above, the collaborative scheme for surface inspection uses two interaction modes between operator and robot: supervised and assisted. Aerostack only supports the first mode with a graphical user interface. Therefore, it was necessary to extend Aerostack to support the second mode. In principle, this extension could be done by adding specialized behaviours for user interaction that request information to the operator through specialized windows. However, as explained below, this solution is not enough, because it is also necessary to extend the way Aerostack coordinates concurrent behaviours to change correctly between the two different interaction modes (supervised and assisted).

**Figure 11:**
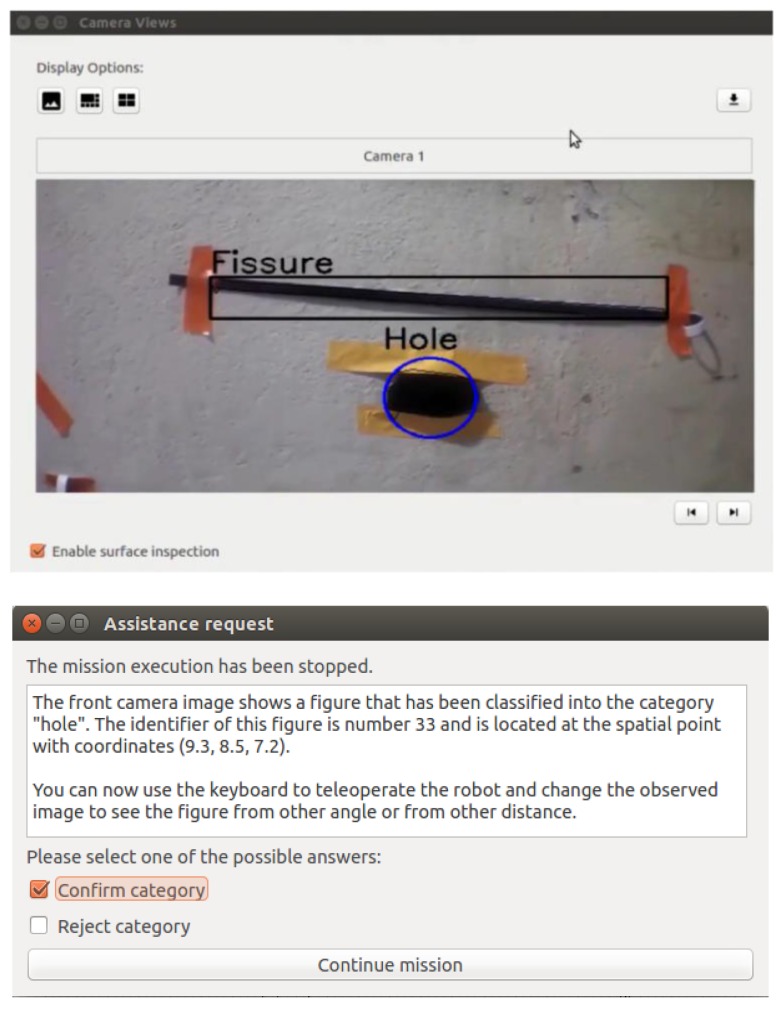
Example of windows to request confirmation of a recognized figure.

To request assistance to the operator, some windows were implemented to present images and texts and wait for the operator answer. For example, Figure 11 shows windows to request confirmation of recognized anomalies. This is implemented with the behaviour *request operator confirm recognition*. The operator can observe the camera image with the camera viewer where the detected imperfections are presented. A pop-up window informs the operator that the mission has been stopped and the type of recognized figure.

If more than one defects are detected, the image highlights in blue colour the figure for which the system is requesting confirmation. The robot keeps a visual tracking of the selected defect until the operator selects one of two options (confirm or reject category) and continue the mission. Meanwhile, the other defects are highlighted in black. The robot memorizes the answer provided by the operator to avoid repeating questions about the same defect. The robot memorizes both the position of the defect (world coordinates) and the operator answer (confirmation or rejection). 

**Figure 12:**
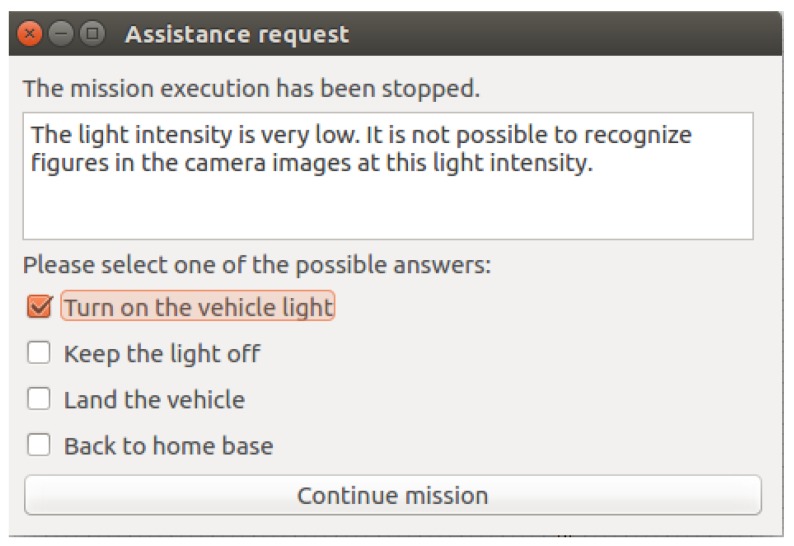
Example of window to request assistance.

Figure 12 shows another type of window to interact with the operator implemented with the behaviour *request operator assistance*. This is a general window that presents different options to the operator and it is useful to use other interaction patterns [42]. As the figure shows, this window can be used by the robot to know how respond in the presence of low light.

As mentioned above, it was also necessary to add another functionality to Aerostack to support the assisted mode. Aerostack provides languages to the operator to specify mission plans (e.g., TML language). During the mission execution, Aerostack interprets the plan written by the operator in these languages to perform the complete mission. This interpretation automatically activates groups of concurrent behaviours according to what is specified in the plan.

The problem is that the assisted mode requires to interrupt the execution of the mission and continue later, after the operator gives an answer. While the mission is paused, the user must be able to execute other behaviours manually, to observe the surface from other angles or distances. This requires stopping part of the active behaviours corresponding to the mission execution, let the operator activate manually other behaviours (e.g., by teleoperation) and finally restore the stopped behaviours to continue the mission.

As a solution for this, Aerostack was extended at the level of behaviour coordination, with a mechanism that informs about the set of active behaviours that are incompatible with a given behaviour. This solution was implemented with a ROS service called *consult incompatible behaviours*. With the help of this, it is possible to pause the execution of a mission to request assistance as it is illustrated by Algorithm 3.
**Algorithm 3.** Assistance request.
1.*B* ← consult incompatible behaviours with the waiting behaviour *b*2.activate the waiting behaviour *b*
3.display window with options *O* = {*o*_1_, *o*_2_, …, *o_n_*}4.*t* ← 05.**repeat**
6.  sleep(*t_s_* seconds) 7.  *t* ← *t + t_s_*8.**until** (operator has selected option *o_i_*) or (*t* > time-out)9.**if** (operator has selected option *o_i_*) **then**
10.  write the result of option *o_i_* in the belief memory11.**for each** *b_j_* **in** *B*
12.  activate behaviour *b_j_*

Let us suppose that when a question is presented to the operator using the corresponding window, we want the robot to stop its movement and keep hovering while waiting for the operator’s response. In general, instead of the behaviour keep hovering, we could use any other behaviour (e.g., align visually at a certain distance in front of the recognized object). Algorithm 3 uses the term *waiting behaviour* to identify this behaviour in a general way.

The first step of the algorithm 3 is to consult the incompatible behaviours with the waiting behaviour. For illustration purposes, let us consider the incompatible behaviour is go to point (3.0, 5.5, 2.5). Then, the waiting behaviour is activated (keep hovering), which stops the movement. This automatically deactivates the incompatible behaviour (go to point). Then, the window that requests information to the operator is displayed, showing alternative options to select {*o*_1_, *o*_2_, …, *o_n_*}. Then, there is a loop to wait until the operator selects one of the options (or until timeout). If the operator selects an option (line 9), the result of this selection is written in the belief memory to be used later during the mission execution. Once this has been done (line 11), the initial behaviours are activated again. In our example, this means that the behaviour go to point (3.0, 5.5, 2.5) is restored to continue the mission.

With this solution, the operation can change consistently between two methods (guided by a mission plan or guided by teleoperation) to support the collaborative scheme described in this paper. This solution is general because it is possible be used by any behaviour that needs to interact with the operator.

## 8. Experimental Evaluation

In order to evaluate our solution, two separate types of tests were applied: (1) tests to evaluate the visual recognition method, and (2) tests to validate the semi-autonomous operation. The following sections describe the details and results of these tests.

### 8.1. Testing the Visual Recognition Method

**Figure 13:**
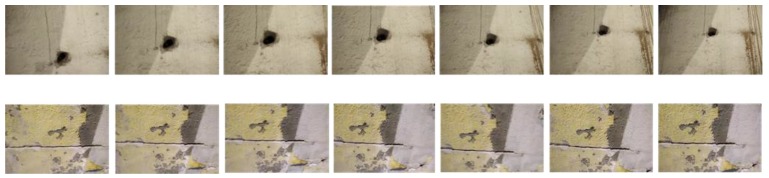
Sample of the dataset of surface imperfections used for the evaluation. The figure presents part of two frame sequences of the video stream take by the camera.

The quality of our method for defect recognition in video streams was evaluated with a dataset of frame sequences. For this purpose, we built a dataset with 650 frames organized in 13 sequences of 50 frames (Figure 13). This dataset is provided as supplementary material of this paper. 

**Figure 14:**
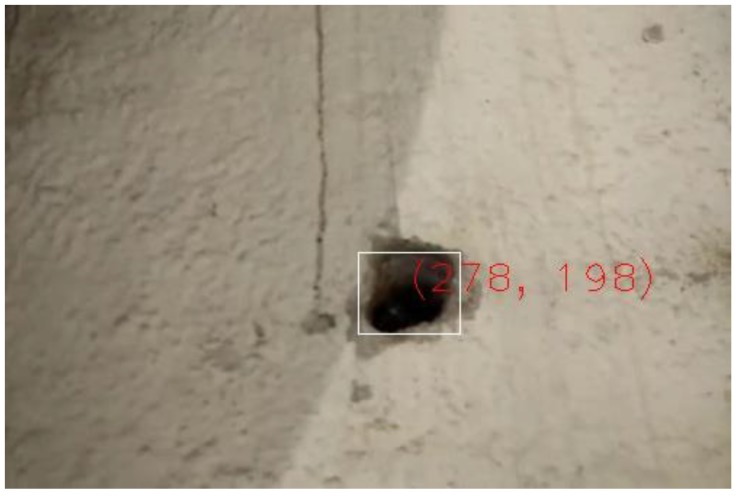
Visualization of data associated to an image of the evaluation dataset.

The dataset includes 6 sequences for holes (3 positive and 3 negative) and 7 sequences for fissures (5 positive and 2 negative). For each positive example, the dataset provides its category (hole or fissure), its size defined by a rectangle covering the figure, and its location (coordinates of the centre of the rectangle in pixels). Figure 14 shows how this information is presented graphically.

The dataset was separated in two parts: (1) a design dataset to estimate the values of parameters {*k*_1_, … *k*_6_, *n*}, and (2) an evaluation dataset to evaluate the quality of the resulting model. The design dataset includes 7 frame sequences and the evaluation dataset 6 frame sequences.

As evaluation metrics, we used precision and recall (and their combination in F-measure). In addition, we also estimated the percentage of times that the recognition method needs operator assistance. The way these values were computed is the following. For precision and recall, true positives are positive examples of the dataset that satisfy *Cr*(*a_j_*) ≥ *k*_5_, false negatives are positive examples of the dataset that satisfy *Cr*(*a_j_*) < *k*_5_, and false positives are negative examples that satisfy *Cr*(*a_j_*) ≥ *k*_6_. To evaluate the level of operator assistance required by the recognition method, we defined a metric called autonomy degree. To obtain the value of this metric, we added the number of examples that satisfy the expression [*Cr*(*a_j_*) < *k*_5_] ∨ [*Cr*(*a_j_*) > *k*_6_] (i.e., the cases when the robot does not require operator assistance) and divided this value by the total number of the examples. 

The design dataset was used to manually calibrate the parameters in order to obtain good values for the evaluation metrics. The parameter values that we found were {*k*_1_ = 0, *k*_2_ = 90, *k*_3_ = 120, *k*_4_ = 11,000, *k*_5_ = 0.2, *k*_6_ = 0.5, *n* = 10}. Table 2 shows the results obtained for the evaluation metrics with the design dataset. We obtained the maximum value for F-measure (1.0) and a value for autonomy degree of 0.96, meaning that the 4% of the examples require operator assistance.

Then, the evaluation dataset was used to analyse the performance of our method using a with-and-without comparison. Two methods were compared:

- *Method without human collaboration*, which corresponds to the FHCE defect recognition method in still pictures described in Section 5.1.
- *Method with human collaboration*, which corresponds to our solution with operator assistance, described in Section 5.2.

The evaluation shows that our solution with human collaboration obtains a high recognition performance (F-measure: 0.98) compared to the other method (F-measure: 0.87) at the cost of reducing the degree of autonomy (0.63) that is, having a partial operator assistance in 27% of the examples. These results are useful to estimate the amount of improvement of the recognition method with the collaborative approach presented in this paper with semi-autonomous operation and partial operator assistance.

### 8.2. Testing the Semi-Autonomous Operation

In addition to the visual recognition tests, other tests were performed to evaluate the capacity of the robot for interaction and autonomous operation. For this purpose, we designed several flight experiments using both simulated flights and real flights. The experiments were designed to validate the correct change between interaction modes (supervised and assisted modes), and the ability to fly efficiently to cover the area to inspect of a surface. 

**Figure 15:**
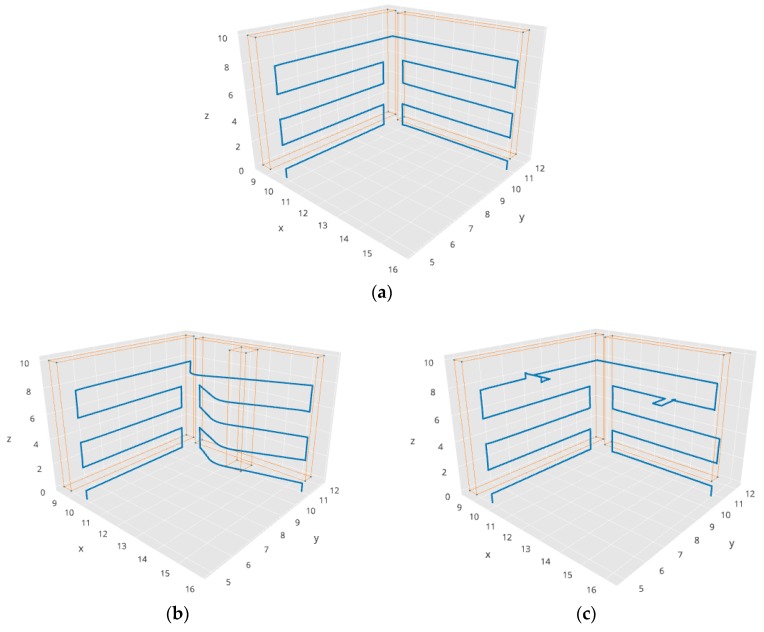
Example trajectories generated by our system (simulated flights). The three figures show the execution of the same mission plan in three different situations. Figure (**a**) shows the trajectory to cover the surfaces defined in the mission plan, figure (**b**) shows how the same plan is adapted when there is an obstacle, figure (**c**) shows two points where the robot stops and asks for assistance to the operator. In the figure, orange lines represent walls and blue lines represent trajectories followed by the aerial vehicle.

For example, in one of the experiments performed by simulation, a surface to inspect with two walls in a corner was defined. Figure 15 shows some of the resulting trajectories generated by our system for the same mission plan in different situations. Figure 15a shows a trajectory to cover the two surfaces of the corner, without obstacles and without interruptions. Figure 15b shows the execution of the same mission, but with a column in one of the walls as an obstacle. In this case, our system adapts correctly the execution of the mission to avoid the obstacle. Figure 15c shows the execution of the same mission, but with two interruptions. The robot stops at these points and asks for assistance to the operator. The operator moves manually (by tele-operation) the robot, answers the question and returns the control to the interpreter of the mission plan to continue the mission execution. 

This experiment proves that the Aerostack language is expressive enough to formulate the movements required for surface inspection and that the robot is able to fly autonomously, correctly following the plan and efficiently describing a trajectory to cover the surface of the walls.

This experiment also verified that the robot was able to interact correctly with the operator to request assistance. Table 3 shows partially the sequence of the behaviour activation. In this experiment, the robot starts the execution guided by a mission plan, that is, the execution is controlled by the plan interpreter that activates behaviours according to what is written in the mission plan. The table shows that the first behaviour to be active is take off. Then, the next behaviours are keep hovering and go to a point with coordinates (9.9, 10.2, 0.7).

After some steps, in step 11, the active behaviour is go to a point with coordinates (9.9, 10.8, 8.4). While the robot is going to this point, the robot recognizes a figure on the wall and automatically activates a behaviour to request assistance to the operator to confirm the recognition (step 12). This behaviour displays a confirmation window to the operator (similar to the window presented in Figure 11) and activates the behaviour keep hovering.

The operator now manually moves the robot by teleoperation (activating other motion behaviours) to observe the surface from other angles or distances. In this case, the operator tele-operates the robot by using the keyboard and activates behaviours such as move up and move backwards to observe the figure from other positions and distances. Finally, the operator confirms the recognition (by clicking the corresponding buttons in the confirmation window) and the behaviour that requests assistance restores the behaviour go to a point with coordinates (9.9, 10.2, 0.7) and returns the control to the plan interpreter to continue the mission.

This type of experiment was repeated with other defects in different positions. The experiments proved that the operation changed consistently between two operation methods (guided by a mission plan or guided by teleoperation) to support correctly the collaborative scheme described in this paper.

**Figure 16:**
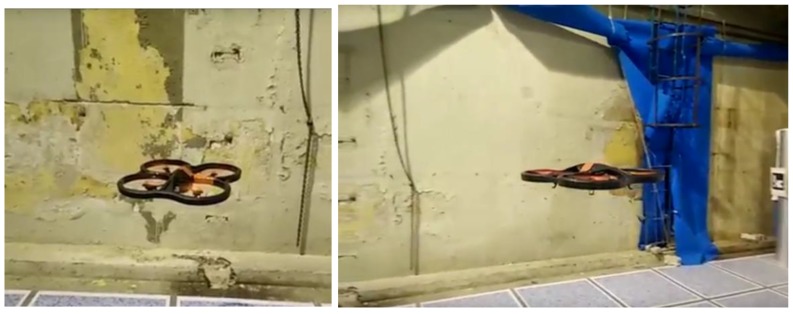
The aerial robot (AR Drone 2.0) during wall inspection.

**Figure 17:**
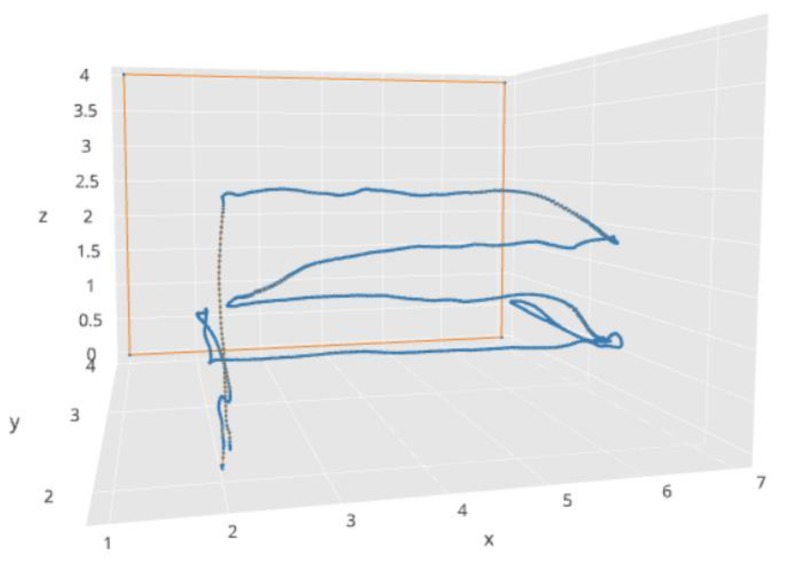
Example exploration trajectory followed by the robot in a real flight. In the figure, the orange line represents the wall and the blue line represents the trajectory followed by the aerial vehicle.

In addition to simulated flights, we also performed tests with real flights. The main goal of these tests was to verify that the robot was able to execute efficiently an inspection plan, doing intermediate stops to have closer views of the wall. We used the platform Parrot AR Drone 2.0 and indoor walls with several imperfections (Figure 16). Figure 17 shows the trajectory developed by the robot in one of the experiments. The robot develops autonomously a horizontal exploration starting from the take-off point (2.0, 2.0, 0.0) and landing at the same point after the exploration. There are two points where the robot zooms in and out to have closer views of the wall: point with coordinates (2.0, 3.0, 1.0) and point with coordinates (6.0, 3.0, 1.0).

In this experiment, the position of the quadcopter was determined by visual odometry. Although this positioning method is not very precise, it was used in the experiments to simplify the practical execution of flight tests. The generated trajectory in this case shows certain irregularities (Figure 17) compared to the ideal trajectories showed by simulations (Figure 15), but this can be improved by using other positioning methods (e.g., with visual markers, lidar, etc.), which is outside the scope of the work presented in this paper. In the experiment, we obtained satisfactory results about efficiency (the whole mission was completed in 1 min and 19 s) and the correct sequence of steps.

## 9. Conclusions

This paper has presented the results of our work on surface inspection using aerial robots with cameras on board. The results show that it is possible to increase the degree of robot autonomy by using an automated visual recognition method together with a human-robot collaboration approach.

In this work, an existing visual recognition method (frequency histogram of connected elements) was extended to be able to operate with partial human assistance. This extension is based on a credibility-based solution that analyses the video stream from the camera to decide when to request assistance to the operator. The evaluation of the assisted method shows a high recognition performance (F-measure: 0.98) compared to a method without assistance (F-measure: 0.87) at the cost of receiving partial operator assistance (in 27% of the cases).

We built a robotic system to validate the type of operation proposed in this paper. The experiments showed that the control changed correctly between different interaction modes (supervised and assisted) and different operation methods (guided by mission plan or by teleoperation) to support the collaborative scheme. 

The software framework Aerostack (www.aerostack.org) was useful to develop our robotic system. This framework provided an architectural pattern that guided the design of our system together with a set of reusable software components to support autonomous behaviour (e.g., motion control, obstacle avoidance, mission plan interpreter, etc.). But it was also necessary to design, implement and integrate new components to support the collaborative approach in surface inspection presented in this paper.

It is also possible to identify potential improvements that could be achieved by further research. For example, our work paid attention to visual recognition and assistance, but the inspection problem includes other autonomous behaviours that can be considered to achieve more autonomy. For example, it would be possible to use elaborated path planning methods to generate automatically the best trajectory to inspect a given surface.

Our visual recognition method has an acceptable performance, according to the goals of a collaborative work, with two types of anomalies (holes and fissures) and vertical surfaces using the frontal camera. Further research can improve the recognition method to increase its performance in order gain more autonomy with less operator assistance and extend its capacity for other types of imperfections (mould, humidity, spots, etc.) together with other camera orientations to cover other types of surfaces.

## Figures and Tables

**Table 1:** Examples of commercial drones with on-board cameras.

Platform	Camera			Flight Time	Range	
	Resolution	Frame Rate	Field of View		Wi-Fi	Transmitter
Parrot AR.Drone 2.0	HD 1280 × 720 px	30 fps	93°	35 min	0 m	
Parrot Bebop 2	FHD 1920 × 1080 px	30 fps	180°	25 min	100 m	2.2 Km
DJI Mavic Pro	UHD 4096 × 2160 px	24 fps	79°	27 min	80 m	7 Km
DJI Phantom 3 Pro	UHD 4096 × 2160 px	30 fps	94°	23 min		5 Km
DJI Inspire 1 Pro	UHD 4096 × 2160 px	30 fps	72°	15 min		5 Km
Yuneec Typhoon H	UHD 4096 × 2160 px	30 fps	94°	25 min	400 m	

**Table 2:** Evaluation results of the recognition methods.

Evaluation Metric	Design Dataset	Evaluation Dataset	
	Method with HC	Method without HC	Method with HC
Precision	1.00	0.93	1.00
Recall	1.00	0.81	0.97
F-measure	1.00	0.87	0.98
Autonomy degree	0.96	1.00	0.63

**Table 3:** Partial sequence of behaviour activation.

Order	Operation Method	Behaviour
01	Guided by mission plan	TAKE_OFF
02	Guided by mission plan	KEEP_HOVERING
03	Guided by mission plan	GO_TO_POINT coordinates: [9.9, 10.2, 0.7]
...	...	...
11	Guided by mission plan	GO_TO_POINT coordinates: [9.9, 10.8, 8.4]
12	Guided by mission plan	REQUEST_OPERATOR_CONFIRM_RECOGNITION
13	Guided by mission plan	KEEP_HOVERING
14	Guided by teleoperation	KEEP_MOVING direction: UP
15	Guided by teleoperation	KEEP_HOVERING
16	Guided by teleoperation	KEEP_MOVING direction: BACKWARDS
...	...	...
20	Guided by teleoperation	KEEP_HOVERING
21	Guided by mission plan	GO_TO_POINT coordinates: [9.9, 10.8, 8.4]
22	Guided by mission plan	KEEP_HOVERING
23	Guided by mission plan	ROTATE angle: 90
...	...	...

## Supplementary Materials

The evaluation dataset and a video illustrating the work presented in this paper are available online at http://www.mdpi.com/1424-8220/18/3/893/s1.
